# Ultrasonic Monitoring of Setting and Strength Development of Ultra-High-Performance Concrete

**DOI:** 10.3390/ma9040294

**Published:** 2016-04-19

**Authors:** Doo-Yeol Yoo, Hyun-Oh Shin, Young-Soo Yoon

**Affiliations:** 1 Department of Architectural Engineering, Hanyang University, 222 Wangsimni-ro, Seongdong-gu, Seoul 04763, Korea; dyyoo@hanyang.ac.kr; 2 Department of Civil Engineering and Applied Mechanics, McGill University, 817 Sherbrooke Street West, Montreal, QC H3A 0C3, Canada; hyunoh777@gmail.com; 3 School of Civil, Environmental and Architectural Engineering, Korea University, 145 Anam-ro, Seongbuk-gu, Seoul 02841, Korea

**Keywords:** ultra-high-performance concrete, setting property, early age tensile strength, ultrasonic pulse velocity, surface treatment

## Abstract

In this study, the setting and tensile strength development of ultra-high-performance concrete (UHPC) at a very early age was investigated by performing the penetration resistance test (ASTM C403), as well as the direct tensile test using the newly developed test apparatus, and taking ultrasonic pulse velocity (UPV) measurements. In order to determine the optimum surface treatment method for preventing rapid surface drying of UHPC, four different methods were examined: plastic sheet, curing cover, membrane-forming compound, and paraffin oil. Based on the test results, the use of paraffin oil was found to be the best choice for measuring the penetration resistance and the UPV, and attaching the plastic sheet to the exposed surface was considered to be a simple method for preventing the rapid surface drying of UHPC elements. An S-shaped tensile strength development at a very early age (before 24 h) was experimentally obtained, and it was predicted by a power function of UPV. Lastly, the addition of shrinkage-reducing and expansive admixtures resulted in more rapid development of penetration resistance and UPV of UHPC.

## 1. Introduction

For several decades, concrete has been widely used in the field of civil and architectural engineering all over the world, because of its excellent mechanical strength, durability, and economic efficiency. However, because of some drawbacks of concrete associated with low tensile strength, low ductility, and low strength-to-weight ratio, its application to thin-plate structures, e.g., thin walls and long-span bridge decks, has been limited thus far. In order to overcome these drawbacks, reactive powder concrete (RPC), which is the forerunner of the currently used ultra-high-performance concrete (UHPC), was developed by Richard and Cheyrezy [1] in the 1990s. Since UHPC is made from a granular mixture optimized by the packing density theory with a low water-to-binder ratio (W/B) and since it includes a high volume content of steel fibers, it exhibits both superior strength and ductility with a unique strain-hardening response.

However, because of the low W/B and the inclusion of high-fineness admixtures in UHPC, the evaporation rate of water from the surface is normally too large to be replenished by bleeding. Because of this, the surface exposed to the atmosphere dries and condenses very quickly, even while most of the interior mortar is still fresh. This causes plastic shrinkage cracks at the surface, as well as durability and aesthetic problems. In addition, much quicker initial and final setting times of UHPC are obtained when these are measured as per ASTM C403 [2], because the needle penetrates through the condensed surface. Therefore, there is a pressing need for new ideas of appropriate methods to prevent the rapid drying and setting of the UHPC surface.

According to a previous study [3], both free and restrained shrinkage of UHPC increase steeply at a very early age, leading to a high possibility of shrinkage cracking. In order to predict the cracking potential precisely, the tensile strength development needs to be investigated along with residual stress resulting from the restraint of shrinkage. For this reason, Yoo *et al.* [4] developed a direct tensile test apparatus to measure the tensile strength before the final set, based on a previous study [5]. However, it is time-consuming to measure the tensile strengths at certain times (especially at a very early age); thus, the field application is limited. An alternative nondestructive method for determining the strength development of concrete has been adopted by several researchers [6,7,8,9,10]. Because of its many advantages (e.g., continuous measurement of microstructural change in concrete, strong relationship between ultrasonic pulse velocity (UPV) and cement hydration, *etc.*), the nondestructive method has attracted much attention from engineers for field applications. However, to the best of the authors’ knowledge, only very limited study [4] is currently available for predicting the tensile strength development of UHPC at a very early age using a nondestructive method.

In order to improve the shrinkage cracking resistance of UHPC, a number of restrained shrinkage tests have been conducted by many researchers [3,11,12,13,14,15]. In particular, Yoo *et al.* [3] reported that the cracking potential can be mitigated by selecting a lower reinforcement ratio and by using a reinforcing bar with a lower stiffness such as a glass fiber-reinforced polymer (GFRP) bar. Park *et al.* [14] employed a ring test to investigate the effect of using shrinkage-reducing admixture (SRA) and expansive admixture (EA) on the restrained shrinkage behavior of UHPC. Based on the test results, they concluded that the combined use of 1% SRA and 7.5% EA exhibits the best performance compared to other mixtures such as a UHPC mixture without SRA and EA or a UHPC mixture with SRA or EA alone. Therefore, the UHPC mixture with 1% SRA and 7.5% EA has been adopted for several structures built in Korea [16]. The effectiveness of the combined use of 1% SRA and 7.5% EA in reducing the shrinkage crack width of UHPC slabs has also been reported by Yoo *et al.* [15].

Accordingly, the present study experimentally investigated the setting, tensile strength, and UPV evolutions of two types of UHPC (with and without SRA and EA) at a very early age (before 24 h). In order to determine the optimum surface treatment method for preventing rapid surface drying, four different methods using plastic sheet, curing cover, membrane-forming compound, and paraffin oil were adopted. In addition, the very early age tensile strength was evaluated by a newly developed tensile test apparatus, and a simple power function relationship was developed to predict tensile strength on the basis of UPV.

## 2. Test Program

### 2.1. Materials, Mixture Proportions, and Mixing Sequence

The mixture proportions used in this study are summarized in Table 1. Type 1 Portland cement produced in Korea and silica fume produced in Norway were used as cementitious materials; their chemical compositions and physical properties are given in Table 2. Silica sand with a grain size smaller than 0.5 mm was used as a fine aggregate, and silica flour including 98% SiO_2_ with a diameter of 2 μm was used as a filler. Coarse aggregate was not included in the mixture to improve its homogeneity. To provide adequate workability and viscosity, 1.2% of a high performance water-reducing agent, polycarboxylate superplasticizer (SP) with a density of 1.06 g/cm^3^, was included. A W/B of 0.2 was adopted, and in order to improve the tensile performance, 2% by volume of short straight steel fibers with a length of 13 mm and a diameter of 0.2 mm were included. The detailed geometrical and mechanical properties of the steel fibers are given in Table 3.

Because of its high autogenous shrinkage and cracking potential at an early age [15], the combined use of 1% SRA and 7.5% EA has been studied by several previous researchers [14,15], and has been adopted for practical applications in Korea [16], as mentioned previously. Therefore, two different mixture proportions for UHPC (*i.e.*, a UHPC mixture without SRA and EA, called “UH-N”, and a UHPC mixture with 1% SRA and 7.5% EA, called “UH-A”) were used in this study, as given in Table 1. Glycol-based SRA (METOLAT P 860) produced by Münzing Chemie GmbH in Heilbronn, Germany and CSA EA produced in Tokyo, Japan (Table 2) were adopted.

For fabricating specimens, a Hobart-type laboratory mixer with 20 L capacity was used. Since UHPC has very low W/B and no coarse aggregate, the mixing sequence was different from that for ordinary concrete. Cement, silica fume, silica flour, and silica sand were premixed for about 10 min. Then, water with SP was added and mixed for another 10 min. When the state of the mortar exhibited proper fluidity and viscosity, the steel fibers were carefully dispersed and then it was mixed for an additional 5 min. Once an adequate fluidity and viscosity for preventing the fiber segregation from the mortar was achieved, the UHPC was cast in a mold. All test specimens were cured in a room with a temperature of 23 ± 1 °C and a humidity of 60% ± 5% during testing.

### 2.2. Test Equipment and Procedure

#### 2.2.1. Penetration Resistance Test (ASTM C403)

In order to investigate the early age setting properties of UHPC, a penetration resistance test was performed as per ASTM C403 [2]. Since the high volume of steel fibers resist the penetration of the needle, a UHPC mixture without steel fiber was used in the present test by simply assuming that the steel fibers have no effect on the setting properties. In addition, very fine silica sand, well dispersed in the mortar, is assumed to have insignificant implications on the setting properties. In the case of UHPC—owing to its low W/B and high fineness admixtures—the water evaporates from the surface faster than it can be replenished by bleeding. Thus, the exposed surface dries rapidly and condenses even while most of the interior cementitious matrix is still fresh. For this reason, if the penetration resistance test is performed without taking into account the rapid surface drying effect, the initial and final setting times will be overestimated. Therefore, in this study, four different surface treatment methods using a plastic sheet, a curing cover, a liquid-type membrane-forming compound, and liquid paraffin oil were adopted, as given in Table 4. Three cylindrical plastic molds with a diameter of 150 mm and a height of 160 mm were used for each variable. The mortar surface was determined by 10 mm below the top edge of the mold to provide the surface treatment methods. The needle penetrated the mortar to a depth of 25 ± 2 mm in 10 s, and the clear distance rule of needle impressions was complied with.

#### 2.2.2. UPV Measurement

**Figure 1:**
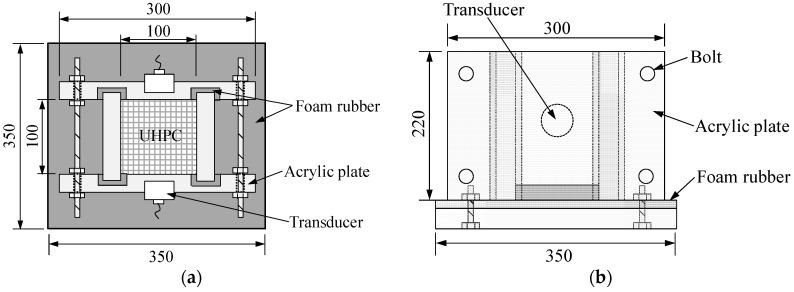
Schematic diagram of UPV monitoring system (unit: mm): (**a**) top view; (**b**) side view.

**Figure 2:**
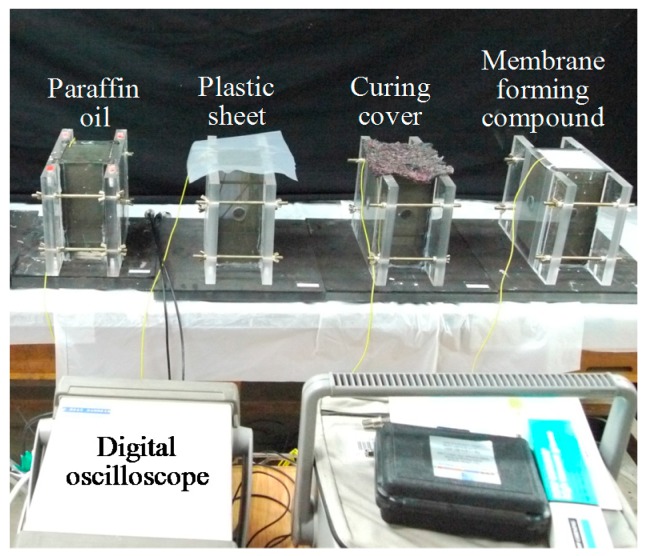
Picture of UPV monitoring system.

Since the microstructural changes of concrete can be consistently estimated by using UPV, several studies [6,7,8,9,10,17,18] have been conducted to examine the early age setting and strength developments in concrete by UPV measurement. In order to measure the UPV immediately after concrete casting, a monitoring system was implemented as shown in Figure 1 and Figure 2.

The mold was composed of four acrylic plates with a thickness of 20 mm, and an acrylic deck. Foam rubber was inserted between the acrylic plates and applied at the bottom surface above the acrylic deck to prevent the propagation of signals through the molds. This is necessary because of the fact that, since UHPC has a very low elastic modulus at an early age, the ultrasonic signals can travel more easily through the acrylic plates than the UHPC matrix. In addition, four bolts were fastened to assure a good contact between the used matrix and the acrylic plates. A commercial device for UPV measurement including a function generator (Agilent 33220A, Santa Clara, CA, USA) for generating the waveform, a filter-amplifier (Krohn-hite 3364, Brockton, MA, USA) for removing random noise and amplifying the received signal, a digital oscilloscope (Agilent 54624A) to measure the signal, and an ultrasonic transducer pair with a nominal frequency of 54 kHz was employed for the UPV measurements [19]. The frequency is applicable for fresh UHPC because its UPV is slower than that of hardened UHPC. Each ultrasonic transducer was mounted inside a circular hole at the mid-height of the mold, and the UPV data was measured by sending a wave from the transmitting transducer into the UHPC matrix and then receiving it with the receiving transducer. The travel time of a wave to pass through the UHPC matrix was recorded by the digital oscilloscope, and the pulse velocity was calculated by

(1) $$V_{p}=L/\Delta t_{p}$$

where *V_p_* is the pulse velocity, *L* is the length of the straight wave path through the specimen, and Δ*t_p_* is the travel time of the wave.

The digital oscilloscope measured only the longitudinal wave velocity. In addition, the measurements started just after concrete casting, and continued for 24 h at 30 min intervals. The steel fibers inside UHPC mortar disturb the accurate measurement of UPV; thus, a UHPC mixture without steel fiber (identical to that used for the penetration resistance test) was adopted.

#### 2.2.3. Direct Tensile Test

**Figure 3:**
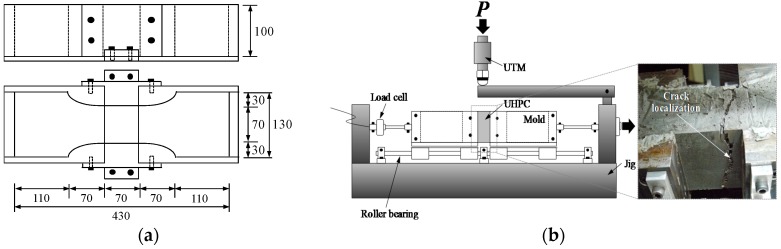
Schematic view of tensile test machine: (**a**) geometry (unit: mm); (**b**) test setup and failure mode.

**Figure 4:**
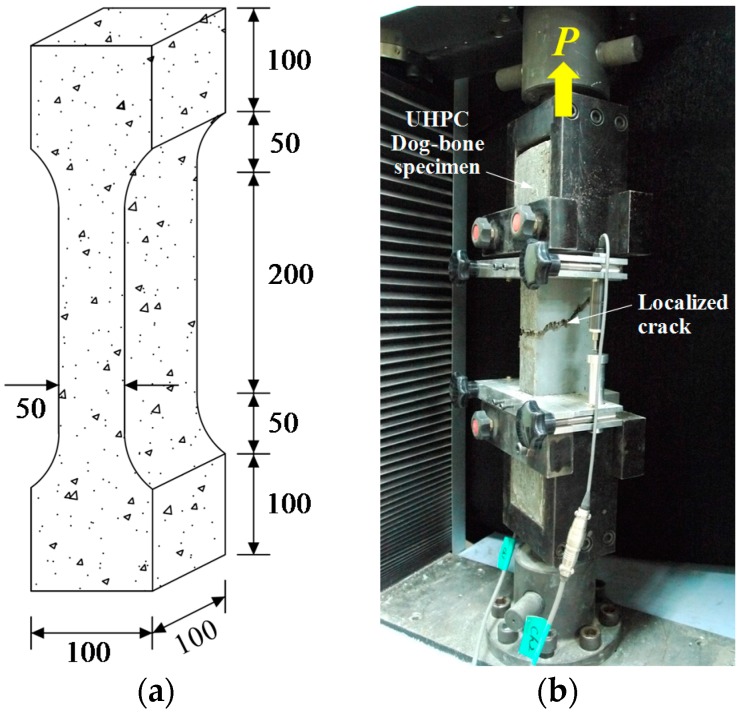
Dog-bone test: (**a**) geometry of specimen (unit: mm); (**b**) test setup.

In accordance with a previous research [5], a direct tensile test apparatus was produced to measure the very early age tensile strength of UHPC. The test apparatus and the geometry of the test mold are shown in Figure 3. A uniaxial load was applied by a universal testing machine (UTM, Shimadzu, Japan) with a maximum load capacity of 250 kN through displacement control at a rate of 0.8 mm/min during testing. Since the tensile strength at a very early age (before 24 h) is extremely low, a load cell with a maximum capacity of 5 kN and a sensitivity of 1.25 N was used up to the tensile strength of 5 kN, and after that, another load cell with a maximum capacity of 20 kN and a sensitivity of 5 N was used. Therefore, reliable data were obtained. When the tensile strength reached almost 2 MPa (shortly after the final set), the tensile strength was measured using a dog-bone test method, as shown in Figure 4 [20]. The cross-section of the dog-bone specimens was 50 mm × 100 mm. The alignment of the test specimen was carefully checked using a plumb before testing. In order to improve the accuracy of the test results, the test setup was designed with so-called pin-fixed ends to avoid secondary flexural stress and to assure the centric loadings. The UTM, identical to that used for very early age tensile strength, was used under displacement control at a rate of 0.8 mm/min.

After concrete casting, all the specimens were immediately covered and adhered to a plastic sheet in order to prevent water evaporation until the testing time.

## 3. Test Results and Discussion

### 3.1. Setting Characteristics of UHPC by Penetration Resistance Test

**Figure 5:**
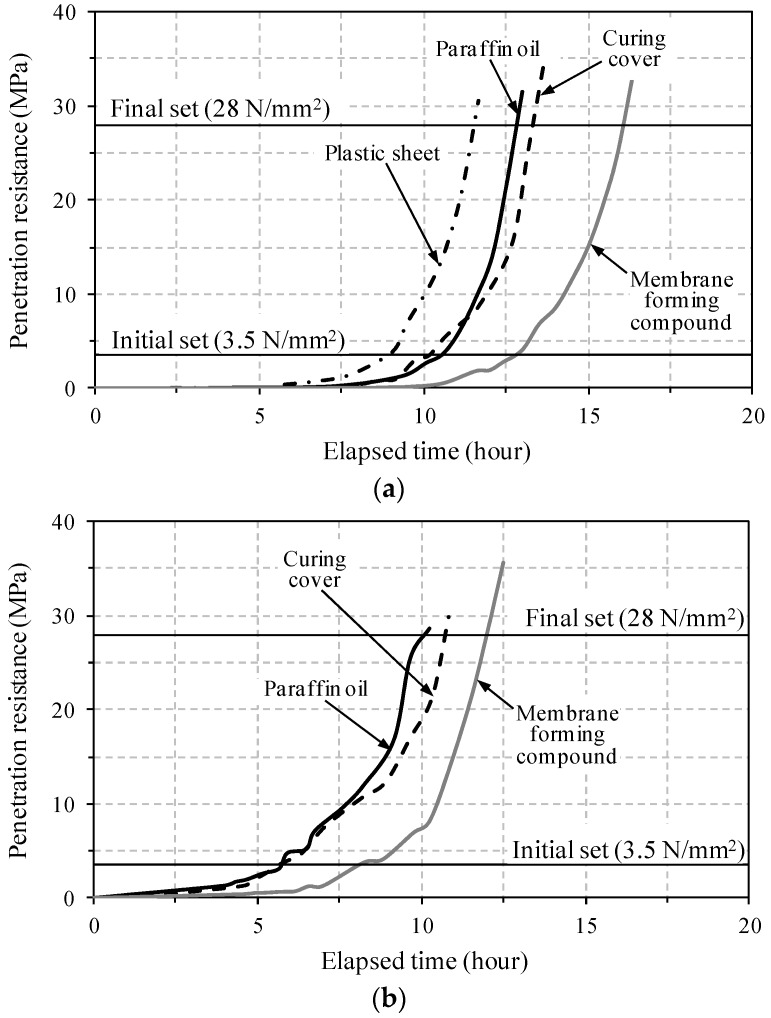
Penetration resistance *versus* elapsed time curve: (**a**) UH-N; (**b**) UH-A.

Figure 5 shows the penetration resistance *versus* elapsed time curves for all test specimens. The development of penetration resistance was noticeably influenced by the surface treatment method. In the case of the plastic sheet, the penetration resistance was developed most rapidly; thus, the initial and final setting times were earlier than those of the other cases in Figure 5a. This is because the plastic sheet was shortly removed from the surface when the needle penetrated, and it caused the rapid surface drying since it was exposed to the atmosphere. This led to an overestimation of the penetration resistance with age. On the other hand, the use of a membrane-forming compound resulted in the slowest development of penetration resistance and the longest initial and final setting times. This is because the membrane-forming compound easily permeated into the UHPC mortar without bleeding, and caused a delay of the setting development. The delay of the strength development and pulse velocity in concrete at an early age has been reported previously by Kim *et al.* [21]. Using a curing cover or paraffin oil resulted in intermediate penetration resistance development and setting times. However, because of the addition of water at the surface, the use of the curing cover delayed the setting development slightly more than the use of paraffin oil. Based on the chemical shrinkage test results performed by Yoo *et al.* [4], the paraffin oil had an insignificant effect on the cement hydration. In addition, since paraffin oil has a lower density (0.88 g/cm^3^) than that of water and does not mix with water, it is able to prevent water evaporation without any obstacle of hydration. Therefore, even though the use of a curing cover produced similar behavior to that of paraffin oil, the use of paraffin oil on the surface was chosen as the most appropriate way for measuring the penetration resistance of UHPC.

The specimen UH-A exhibited more rapid development of penetration resistance with age, causing earlier initial and final setting times, compared to the specimen UH-N without EA and SRA. This is consistent with the findings from a previous study [15], and is caused by fact that the development of setting in UHPC mortar was accelerated by the formation of large quantities of ettringite at an early age, resulting from the use of EA. On the other hand, a more gradual increase of penetration resistance after the initial set was obtained for UH-A than that for UH-N. The measured initial and final setting times according to the surface treatment method are summarized in Table 5.

### 3.2. Evolution of UPV

**Figure 6:**
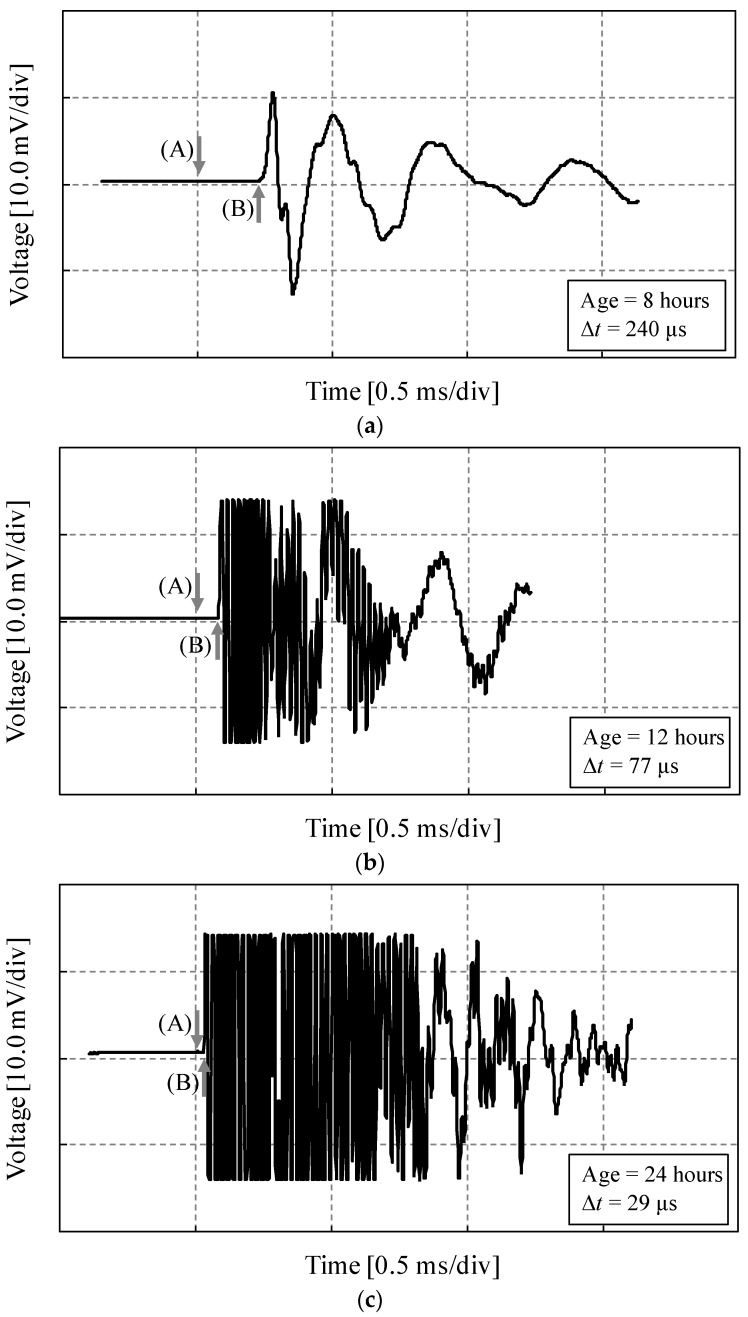
Ultrasonic waveforms from oscilloscope for UH-N with paraffin oil: (**a**) 8 h; (**b**) 12 h; (**c**) 24 h.

The typical ultrasonic waveforms of UH-N with paraffin oil at three different ages are shown in Figure 6. The waveforms were measured by recording the voltage signal at the receiving transducer with an oscilloscope (Figure 2). Point (A) with the downward arrow indicates the time when the input pulse was first transmitted. Point (B) with the upward arrow indicates the time when the ultrasonic wave was received by the receiving transducer. Because of the increase of stiffness in the UHPC mortar with age, the travel time of the ultrasonic wave was noticeably reduced as the mortar aged. In addition, a much higher dominant frequency in the waveforms was obtained at the ages of 12 and 24 h, compared to that of 8 h. This is because the stiffness (penetration resistance) of the mortar steeply increased after about 8 h, as shown in Figure 5a; thus, the pulse velocity through the mortar substantially increased from that point.

**Figure 7:**
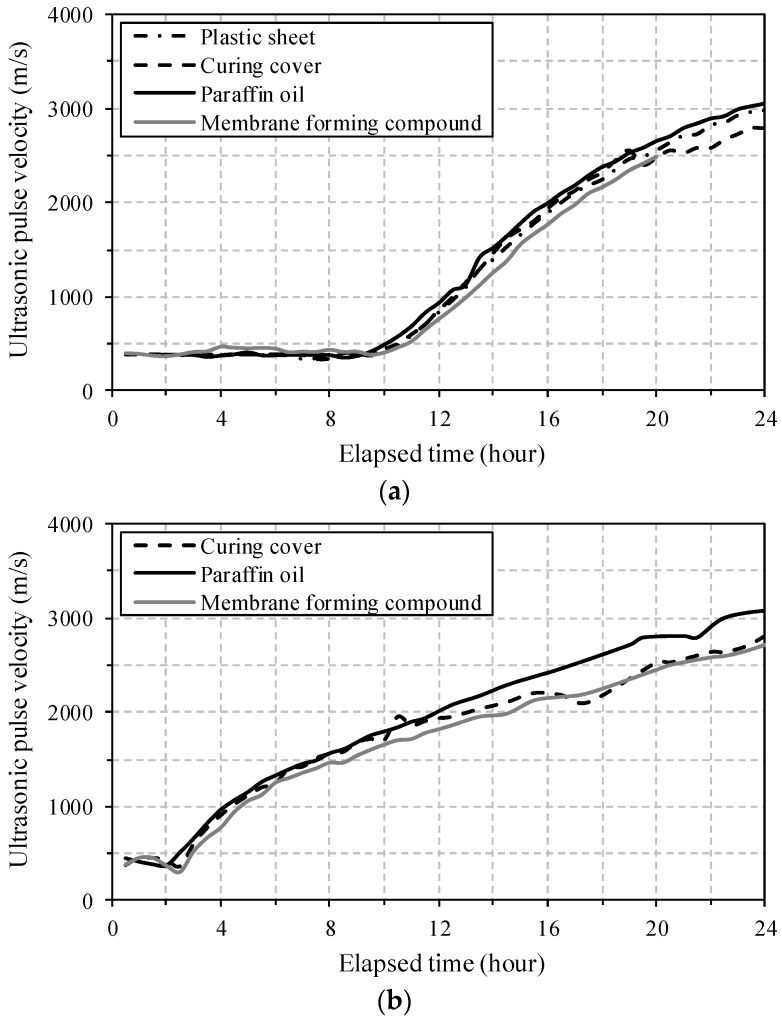
Evolution of UPV in UHPC mortar: (**a**) UH-N; (**b**) UH-A.

The travel time of the ultrasonic wave in Equation (1) was obtained by subtracting the transit time of the ultrasonic wave through the acrylic plates from the measured total time. Figure 7 shows the evolution of UPV calculated by Equation (1) during the first 24 h. The shape of UPV development was not significantly affected by the surface treatment method, whereas the UPV values at certain ages (after an increase point) were slightly lower when a curing cover or membrane-forming compound was used compared to the values when paraffin oil was used. This is consistent with the results of the penetration resistance test in Figure 5. However, the use of a plastic sheet, which adhered to the exposed surface immediately after casting, provided similar UPV values as those of paraffin oil, which is inconsistent with the findings from the penetration resistance test. This is because the plastic sheet was not removed from the surface during UPV testing, in contrast to the case of penetration resistance testing. Therefore, it is noted that the plastic sheet is effective for preventing rapid water evaporation at the surface of the UHPC samples (*i.e.*, mechanical test samples, structural elements, *etc.*).

UPVs slightly decreased at a very early age, because when the cement was hydrated with water, the air-filled space in the cement paste increased [22]. This observation was more obvious in the specimen UH-A than UH-N. Note in Figure 7 that specimen UH-A clearly exhibits an earlier starting time for the UPV increase than its counterpart. This is caused by the accelerated setting development at an early age, which, in turn, is caused by the large quantities of ettringite resulting from the addition of EA. On the other hand, a more gradual increase of the UPV was observed for specimen UH-A than for UH-N, which is consistent with the penetration resistance test results. Similar UPV values at 24 h were obtained for both UH-N and UH-A.

The values of UPV at 24 h obtained in this study (approximately 3000 m/s) were quite similar to the UPVs of ordinary cement mortar at 24 h, reported by Reinhardt *et al.* [17]. Specifically, the UPVs corresponding to the initial and final setting times (in Table 5) were found to be 685 m/s and 1217 m/s for UH-N, and 1276 m/s and 1804 m/s for UH-A, respectively, for the case when paraffin oil was used. Thus, these UPV values can be used for determining the initial and final setting points of UHPC.

**Figure 8:**
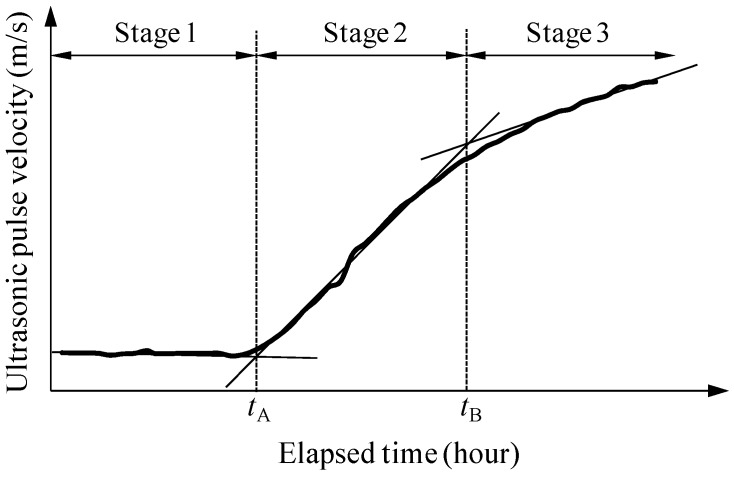
Schematic view of UPV development (UH-N with paraffin oil).

Figure 8 shows a schematic view of typical UPV evolution. In agreement with previous research [17,18,23], an S-shaped UPV development was obtained. In stage 1, the ultrasonic wave propagated through the fresh UHPC mortar (a water-like viscous phase), with a relatively low UPV of approximately 400 m/s. At the onset of the hydration process, a slight decrease of UPV was obtained, resulting from the increase of air-filled pores [22]. As the hydration continued, a minimum quantity of hydration products was formed and then the UPV increased sharply (stage 2). The water-saturated porous solid structure was continuously connected by the hydration products; thus, the waves propagated through the solid matrix instead of through the water-like viscous phase. Accordingly, a noticeable rate of increase of the UPV was observed during stage 2. Once most of the pores were filled by hydration products, the slope of the UPV increase gradually decreased and the UPV converged to a constant value in the solid structure (stage 3).

In the case when paraffin oil was used, the inflection points *t*_A_ and *t*_B_ in the S-shaped UPV evolution curves are summarized in Table 6. The specimen UH-A exhibited a much shorter time to complete stage 1, owing to the acceleration of stiffness development in the mortar that included EA. In the same manner, a faster initiation of the steep increase in the shrinkage was obtained for UH-A (with 1% SRA and 7.5% EA), compared to that for UH-N (without SRA and EA) [13].

### 3.3. Early Age Tensile Strength Development

**Figure 9:**
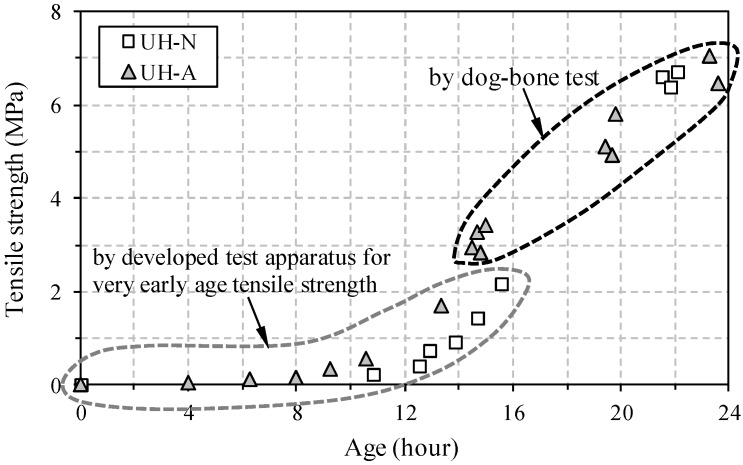
Early age tensile strength development (before 24 h).

The tensile strength development at a very early age is illustrated in Figure 9. Just as with the UPV evolution, S-shaped curves were obtained for both UH-N and UH-A. Before the tensile strength reached approximately 2 MPa (at an applied load of approximately 14 kN), the developed tensile test apparatus was used (Figure 3), and after that, the dog-bone test method was adopted for measuring the tensile strength (Figure 4). Specimen UH-A exhibited higher tensile strength at a very early age than UH-N did; this is consistent with the findings from the results of the penetration resistance test and the UPV measurements. On the other hand, the tensile strengths became similar to each other at about 24 h, which is the same trend that was observed in the case of UPV evolution. For example, the average tensile strengths were found to be 6.56 MPa for UH-N and 6.45 MPa for UH-A at about 22 h.

In order to predict the very early age tensile strength of UHPC with respect to UPV, the simple power function previously suggested by Pessiki and Carino [6] was adopted in this study, as follows:

(2) $$f_{t}=a{\left(V_{p}\right)}^{b}$$

where *f_t_* is the tensile strength (MPa), *V_p_* is the pulse velocity (km/s), and *a* and *b* are the coefficients. In the current study, Equation (2) was chosen because it has a simple formulation and exhibits the best fit with the experimental results compared to the other models suggested by Pessiki and Carino [7] and Pessiki and Johnson [9], which are most widely used at the present time.

**Figure 10:**
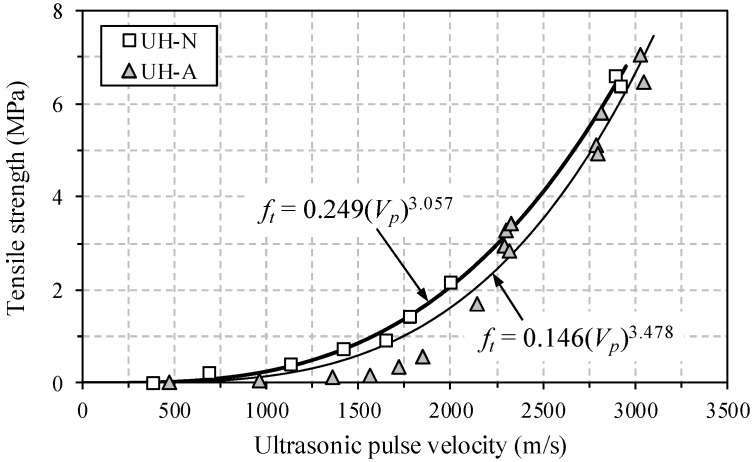
Relationship between early age tensile strength and UPV.

The comparison of tensile strength and UPV is shown in Figure 10. The tensile strength was increased with the UPV in exponential or power function form. This is consistent with the findings from Pessiki and Carino [7] and Keating *et al.* [8] for the relationship between compressive strength and UPV. In addition, the relationship between tensile strength and UPV at a very early age was accurately predicted using a simple power function, *i.e.*, the coefficient of determination (*R*^2^) was found to be 0.99691 for UH-N and 0.96742 for UH-A.

## 4. Conclusions

This study investigated the effects of the surface treatment method on the setting properties and UPV evolution of two types of UHPC. In addition, very early age tensile strength development (before 24 h) was measured by a newly developed tensile test apparatus and correlated with the UPV. From the above discussions, the following conclusions can be drawn:

(1) The use of a plastic sheet caused an overestimation of the penetration resistance, whereas the addition of a membrane-forming compound led to an underestimation. The use of either paraffin oil or a curing cover resulted in intermediate values with behaviors that were similar to each other. However, the paraffin oil was chosen to be most appropriate for measuring the penetration resistance of UHPC because of its lower density (0.88 g/cm^3^), its immiscibility with water, and the fact that it did not interfere with hydration.

(2) The shape of UPV development with age was not affected by the surface treatment method. In contrast, the use of a curing cover or membrane-forming compound resulted in slightly lower UPV values at certain ages, compared to the results when paraffin oil was used. Attaching a plastic sheet to the exposed surface was effective at preventing water evaporation, and thus, it is considered to be a simple method for preventing the rapid surface drying of UHPC elements.

(3) The specimen UH-A (with 1% SRA and 7.5% EA) exhibited more rapid development of penetration resistance, leading to quicker initial and final setting times, and an earlier starting time for the steep increase in the UPV evolution, compared to those of UH-N (without SRA and EA). This is mainly caused by the accelerated stiffness development in the UHPC mortar at an early age due to the large quantities of ettringite that result from the addition of EA.

(4) The S-shaped tensile strength development of UHPC at a very early age (before 24 h) was successfully measured in this study. The tensile strength increased monotonically with the UPV values and could be accurately predicted by using a simple power function.

## Figures and Tables

**Table 1:** Mixture proportions of UHPC (unit weight, kg/m^3^).

Specimen	Water	Cement	Silica Fume	Silica Flour	Silica Sand	EA	SRA	SP (%)	Steel Fiber (*v_f_*, %)
UH-N	160.3	788.5	197.1	236.6	867.4	-	-	1.2	2.0
UH-A	165.5	786.6	196.7	236.0	865.3	59.0	7.9	1.2	2.0

Note: EA = expansive admixture, SRA = shrinkage-reducing admixture, and SP = superplasticizer.

**Table 2:** Chemical compositions and physical properties of cementitious materials and admixture.

Composition % (Mass)	Cement	Silica Fume	Expansive Admixture
CaO	61.33	0.38	13.55
Al_2_O_3_	6.40	0.25	18.66
SiO_2_	21.01	96.00	3.80
Fe_2_O_3_	3.12	0.12	-
MgO	3.02	0.10	-
SO_3_	2.30	-	51.35
K_2_O	-	-	0.56
F-CaO	-	-	16.02
Specific surface (cm^2^/g)	3413	200,000	3117
Density (g/cm^3^)	3.15	2.10	2.98

**Table 3:** Properties of straight steel fibers.

Diameter *d_f_* (mm)	Length *L_f_* (mm)	Aspect Ratio (*L_f_*/*d_f_*)	Density (g/cm^3^)	Tensile Strength (MPa)	Elastic Modulus (GPa)	Image
0.2	13.0	65.0	7.9	2500	200	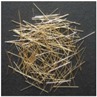

**Table 4:** Surface treatment methods on casting surface.

Specimen	Plastic Sheet	Curing Cover	Membrane Forming Compound	Paraffin Oil
UH-N	O	O	O	O
UH-A	X	O	O	O

**Table 5:** Initial and final setting times.

Specimen	Surface Treatment	Initial Set (Hour)	Final Set (Hour)
UH-N	Plastic sheet	8.8	11.5
	Curing cover	10.2	13.2
	Membrane forming compound	12.8	16.0
	Paraffin oil	10.5	12.8
UH-A	Curing cover	5.6	10.7
	Membrane forming compound	8.0	11.9
	Paraffin oil	5.8	10.2

**Table 6:** Inflection points in UPV evolution curves.

Specimen	*t_A_* (Hour)	*t_B_* (Hour)
UH-N	9.8	17.2
UH-A	2.0	5.1
